# Studying Familial Bainbridge–Ropers Syndrome Due to a Novel *ASXL3* Germline Variant and Expanding the Clinical Spectrum

**DOI:** 10.3390/children13050599

**Published:** 2026-04-27

**Authors:** Daiana Mariano, Valentina Petrone, Francesca Madia, Mariasavina Severino, Luca Basso, Valeria Capra, Maria Stella Vari, Antonio Marras, Giacomo Tantari, Giuseppe d’Annunzio

**Affiliations:** 1 Department of Neuroscience, Rehabilitation, Ophthalmology, Genetics, Maternal and Child Health (DINOGMI), University of Genoa, 16132 Genoa, Italy; daiana.mariano@edu.unige.it (D.M.); 6554066@studenti.unige.it (V.P.); 2 Medical Genetics Unit, IRCCS Istituto Giannina Gaslini, 16147 Genoa, Italy; francescamadia@gaslini.org; 3 Neuroradiology Unit, IRCCS Istituto Giannina Gaslini, 16147 Genoa, Italy; mariasavinaseverino@gaslini.org; 4 Radiology Unit, IRCCS Istituto Giannina Gaslini, 16147 Genoa, Italy; lucabasso@gaslini.org; 5 Clinical Genomics and Genetics Unit, IRCCS Istituto Giannina Gaslini, 16147 Genoa, Italy; valeriacapra@gaslini.org; 6 Pediatric Neurology and Muscular Disease Unit, IRCCS Istituto Giannina Gaslini, 16147 Genoa, Italy; mariastellavari@gaslini.org; 7 Pediatric Clinic and Endocrinology Unit, IRCCS Istituto Giannina Gaslini, 16147 Genoa, Italy; antoniomarras@gaslini.org (A.M.); giuseppedannunzio@gaslini.org (G.d.)

**Keywords:** *ASXL3* gene, *ASXL3*-related syndrome, Bainbridge–Ropers syndrome, BRPS, neurodevelopmental disorder

## Abstract

**Background/Objectives**: Bainbridge–Ropers syndrome (BRPS) is a rare neurodevelopmental disorder caused by truncating and splicing pathogenic variants in the additional sex combs-like 3 (*ASXL3*) gene. It is primarily characterized by neurodevelopmental delay and craniofacial dysmorphism. Most reported cases involve de novo mutations in the *ASXL3* gene, whereas inherited mutations have been rarely described. The present report aims to describe the clinical and molecular presentation of a familial case of BRPS and to highlight the potential role of parental mosaicism. **Methods**: We describe the clinical and molecular presentation of a 12-year-old boy and his 20-month-old half-brother, both affected by Bainbridge–Ropers syndrome. Trio-exome sequencing (ES) was performed in the family to identify variants in the *ASXL3* gene, and targeted Sanger sequencing was also performed for segregation analysis. **Results**: Genetic analysis identified a previously unreported heterozygous frameshift variant in the *ASXL3* gene (c.1648_1649del; p.Met550Aspfs*5) shared by both siblings. The variant was inherited from their clinically unaffected mother, who carries the mutation in the mosaic state with a variant allele fraction of approximately 15% in peripheral blood DNA. **Conclusions**: This observation highlights parental mosaicism as a potential mechanism underlying the familial recurrence of BRPS and emphasizes the importance of considering mosaic variants during the genetic evaluation and counseling of affected families.

## 1. Introduction

Bainbridge–Ropers syndrome (OMIM ID: 615485) is a rare neurodevelopmental disorder first described in 2013 by Bainbridge et al. in four unrelated children presenting similar phenotypic features [1]. Whole-exome sequencing revealed de novo truncating pathogenic variants in the ASXL3 gene in all four individuals. Since then, de novo mutations in the ASXL3 gene have predominantly been reported as causative of BPRS [2,3,4,5,6,7,8,9,10,11], and familial occurrence was first described by Schirwani et al. in 2021 [12]. Both de novo and inherited cases of BRPS exhibit significant phenotypic heterogeneity, and intrafamilial phenotypic variations have been poorly explored [13,14,15].

The *ASXL* gene family includes three members—*ASXL1*, *ASXL2*, and *ASXL3*—which are human homologs of the *Drosophila* gene additional sex combs (*ASX*) and encode proteins involved in transcriptional regulation. Specifically, *ASXL3* interacts with BAP1, forming a key component of the PR-DUB (polycomb repressive deubiquitinase) complex, which removes mono-ubiquitin from Histone H2A lysine 119 (H2AK119Ub1) [16,17].

To explore the molecular consequences of *ASXL3* gene loss-of-function variants, Srivastava et al. analyzed primary dermal fibroblasts derived from a BRPS patient carrying a de novo truncating mutation and demonstrated that the mutant transcripts are subject to nonsense-mediated decay, resulting in markedly reduced *ASXL3* gene expression, impaired PR-DUB activity and consequently increased levels of H2AK119Ub1 [17]. The altered expression of genes influencing transcriptional regulation, development and proliferation has been observed in fibroblasts from BRPS patients [17].

Somatic mutations of all three *ASXL* gene family members are reported in human cancers [18,19], and germline mutations have been associated with clinically distinct but overlapping syndromes: pathogenic variants in the *ASXL1* gene were found in Bohring–Opitz Syndrome (BOPS), in the *ASXL2* gene in Shashi–Pena Syndrome (SHAPNS) and in the *ASXL3* gene in Bainbridge–Ropers syndrome [19].

Bainbridge–Ropers syndrome is characterized by global developmental delay; feeding difficulties; failure to thrive; hypotonia; language impairment, ranging to the absence of speech; moderate to severe intellectual disability (ID); autistic traits and dysmorphic features [20,21,22]. Characteristic craniofacial features include a prominent forehead, down-slanting palpebral fissures, prominent nasal bridge, broad nasal tip, low columella, high-arched palate, and crowded teeth.

Because the clinical presentation of ASXL3-related disorders is typically non-specific global developmental delay, all disorders associated with intellectual disability should be considered in the differential diagnosis.

Furthermore, no distinctive phenotypic features are related to BPRS [21], and generally, access to molecular studies for individuals with intellectual disability remains extremely limited, resulting in a significant underdiagnosis of BPRS. Although some pathogenic variants have been recently associated with specific features [13], no clear genotype–phenotype correlation has been established [23,24,25]. In a study published in January 2025, a syndrome phenotype was characterized in a cohort of 57 individuals carrying pathogenic *ASXL3* gene variants [25]. The authors reported 24 novel variants, including six families with inherited *ASXL3* gene mutations, and for the first time, specific management recommendations were outlined. These include the early initiation of therapeutic interventions, ophthalmological and dental follow-up, and a baseline renal ultrasound [25].

To date, approximately ten cases of ASXL3-related syndrome have been documented in the literature, wherein trio-exome sequencing confirmed the parental inheritance of the pathogenic variant. Notably, in three of these instances, the syndrome was inherited from a mosaic parent [13,14,15].

Here we describe a family with inherited BRPS, consisting of two maternal half-brothers with a newly identified truncating mutation of the *ASXL3* gene and their mother who is mosaic for the same variant. These findings further support the heritability of the syndrome and underscore its phenotypic heterogeneity, including intrafamilial variability [26].

## 2. Materials and Methods

We describe two patients and their mother who were admitted via the emergency department to the Pediatric Clinic and Endocrinology Unit of the IRCCS Istituto Giannina Gaslini, Genoa, Italy, where comprehensive clinical, diagnostic and molecular investigations were performed. The diagnostic workup included clinical and neurological assessments (Bayley Scales of Infant and Toddler Development, Third edition; Pearson San Antonio, TX, USA; and Griffiths Mental Development scales, third edition; Hogrefe, Oxford, UK), pelvic X-ray, esophageal transit X-ray, electroencephalogram (EEG), brain and spine Magnetic Resonance Imaging (MRI), abdominal ultrasound (US) with echo–color Doppler, abdominal MRI, Next-Generation Sequencing (NGS) and Sanger sequencing. A multidisciplinary team including pediatricians, endocrinologists, geneticists, neurogeneticists, child neuropsychiatrists, neuroradiologists and radiologists was involved in the evaluation. All procedures were conducted in accordance with ethical standards, and written informed consent was obtained from the parent.

### Molecular Diagnosis

For genetic testing, trio-exome sequencing was performed in the family on genomic DNA extracted from peripheral blood. The coding genomic region and flanking intronic sequences (exome) were selected through the SOPHia Whole Exome Solution Kit, Gene Panel WES_v1 (40.9 Mb, 20.133 genes; SOPHiA GENETICS SA, Saint-Sulpice, Switzerland). ES was performed using two analysis platforms: an in-house bioinformatics pipeline based on Burrows-Wheeler Aligner (BWA, version 0.7.17) and Genome Analysis Toolkit (GATK, version 4.2; Broad Institute, Cambridge, MA, USA) following GATK Best Practices (https://gatk.broadinstitute.org/; accessed on 7 September 2025), and a commercial platform (Sophia DDM^TM^, version 4; SOPHiA GENETICS SA, Saint-Sulpice, Switzerland). Targeted Sanger sequencing using standard methods was also performed for both the verification of identified variants and segregation analysis. After filtering for allele frequency (≤0.01% in public databases, including Genome Aggregation Database, gnomAD v2.1.1; Broad Institute, Cambridge, MA, USA; https://gnomad.broadinstitute.org/; accessed on 7 September 2025). Candidate variants were screened according to family segregation, conservation (GERP score), predicted impact on protein function using in silico tools (SIFT, PolyPhen-2, MutationTaster, AlphaMissense), and presence in clinical databases (ClinVar; Narional Center for Biotechnology Information, Bethesda, MD, USA). Candidate variants were classified using Franklin (Genoox Ltd., Tel Aviv, Israel; https://franklin.genoox.com/; accessed 7 September 2025) according to the American College of Medical Genetics and Genomics criteria [27]. *ASXL3* gene variants are listed according to the transcript NM_030632 and referenced to the hg38/GRCh38 assembly.

## 3. Case Presentation

### 3.1. Proband 1

In this case, the patient is a 12-year-old boy, who was born in Albania at 36 weeks of gestation via spontaneous vaginal delivery, to non-consanguineous parents, including a 24-year-old mother, with a birth weight of 1600 g (−2.48 SDS, 1st percentile), classified as small for gestational age (SGA). The pregnancy was unmonitored and notably marked by exposure to physical abuse.

There was a family history of neuropsychiatric disease (maternal uncle with psychosis and depression) and developmental delay (20-month-old maternal half-brother).

At birth, he was admitted to neonatal intensive care because of hypotonia and hyperbilirubinemia. At 15 months, he developed epilepsy and started therapy with Valproic Acid and Levetiracetam, which resulted effective at the standard therapeutic dose (Valproic Acid 30 mg/kg/die and Levetiracetam 3 mg/die). The type and dynamics of the seizures were not specified in previously available medical reports nor were the characteristics of the investigations performed (e.g., type of EEG and brain MRI). At 5 years of age, his neurological follow-up was interrupted: the last EEG showed widespread paroxysmal abnormalities, and brain MRI was reported as negative. Psychomotor development was significantly delayed, with independent walking achieved at 4 years of age. Language development was limited to the first words at 2 years, followed by regression, and the patient is currently nonverbal. Sphincter control was never attained.

At 12 years of age, he was eventually brought to the emergency department of our hospital from abroad and subsequently admitted to the Pediatric Clinic and Endocrinology Unit, IRCCS Istituto Giannina Gaslini, Genoa. He showed developmental delay and neuropsychiatric disease (epilepsy, inverted sleep–wake rhythm and psychomotor agitation), microcephaly and dysmorphic features. Due to the severity of the psychomotor delay, formal neuropsychological testing could not be performed. On physical examination, craniofacial dysmorphism was reported, including microcephaly, left ptosis, a small lipoma on the left frontal area, sparse and thin eyebrows, prominent columella, hypoplasia of the nasal alae, malocclusion and dental overcrowding, and hypertrophic tragus. Auxological evaluation showed a weight of 34 kg (>5th percentile according to Centers for Disease Control and Prevention (CDC). CDC Growth Charts: United States, 2000. Available online: https://www.cdc.gov/growthcharts/, accessed on 7 September 2025), an occipitofrontal circumference of 51 cm (−2 SDS) and pre-pubertal development. Height could not be accurately measured because of psychomotor agitation.

**Figure 1:**
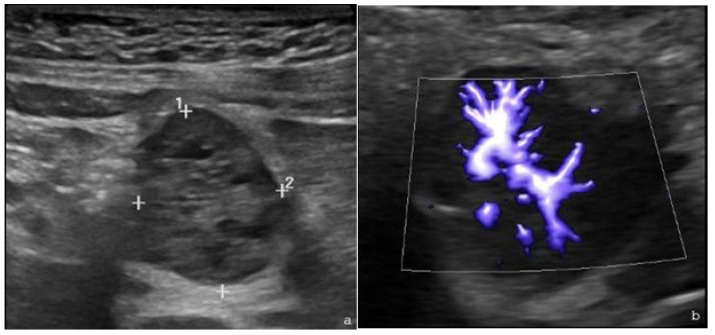
Abdominal US with echo–color Doppler in proband 1. (**a**) Abdominal B-mode US reveals a solid inhomogeneous hypoechoic formation with maximum dimensions 2.4 × 1.9 cm adjacent to the gallbladder and the left liver lobe. (**b**) Echo–color Doppler microvascular imaging (MVI) depicts the vascular hilum. The “+” symbols represent the ultrasound calipers used for lesion measurements. The accompanying numbers correspond to point identifiers and do not indicate lesion size.

**Figure 2:**
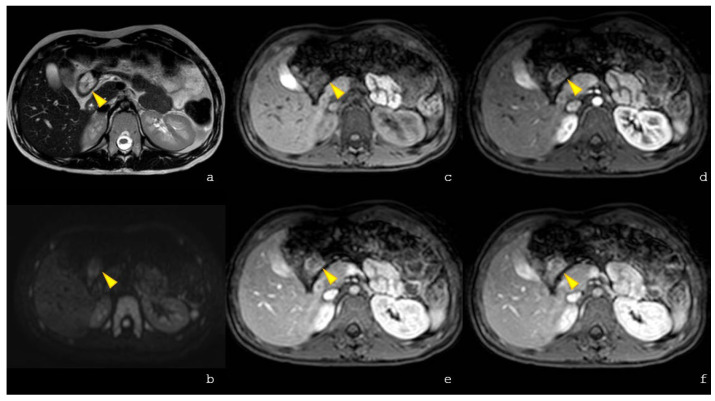
MRI examination in proband 1: Axial T2-weighted imaging (**a**) confirms the presence of a hyperintense nodular formation with well-defined margins, localized within the colonic lumen at the hepatic flexure indicated by the yellow arrowhead. The lesion shows no restricted diffusion (**b**), is characterized by no hyperintense signal intensity on pre-contrast axial eTRHIVE images (**c**) and shows early contrast enhancement during the arterial phase (**d**), persisting in subsequent phases (**e**,**f**). Coronal SPAIR T2-weighted images show the presence of a parapelvic cyst (diameter 11 mm) at the middle of the right kidney (**e**) and cortical cyst (diameter 5 mm) at the lower pole of the homolateral kidney (**f**).

**Figure 3:**
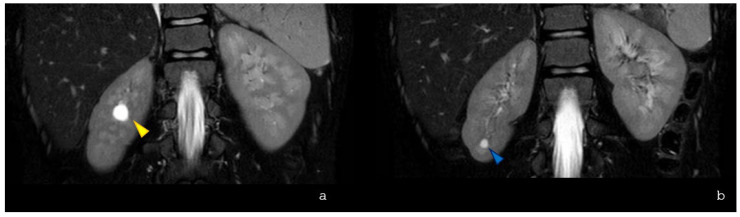
Coronal SPAIR T2-weighted images show the presence of a parapelvic cyst (diameter 11 mm) at the middle of the right kidney (**a**) [yellow arrowhead] and cortical cyst (diameter 5 mm) at the lower pole of the homolateral kidney (**b**) [blue arrowhead].

The patient showed decreased appetite and fever; therefore, an abdominal US with echo–color Doppler was performed, revealing a hypoechoic formation at the epigastrium, adjacent to the left lobe of the liver, with a vascular hilum (Figure 1a,b). Abdominal MRI confirmed a well-defined nodular lesion at the colonic hepatic flexure and hyperintensity on T2-weighted imaging with early arterial-phase contrast enhancement persisting in later phases (Figure 2). The incidental findings included two simple right renal cysts (Figure 3).

Repeated fecal blood testing was negative. A colonoscopy revealed a pedunculated polypoid lesion with a violet- and red-spotted surface, whose histological examination led to the diagnosis of juvenile polyp.

**Figure 4:**
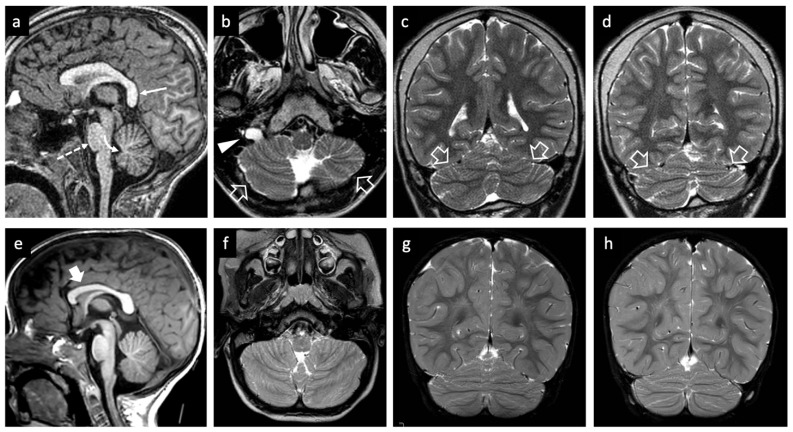
Brain MRI studies of proband 1 at 12 years of age (**a**–**d**) and of proband 2 at 1 year and 9 months (**e**–**h**). (**a**) The sagittal T1-weighted image reveals a vertical splenium (arrow) associated with a small pons (dashed arrow) and hypoplastic vermis with low-lying fastigium (curved arrow). (**b**–**d**) Axial (**b**) and coronal (**c**,**d**) T2-weighted images demonstrate a smaller left cerebellar hemisphere with bilateral abnormal orientation and depth of the cerebellar sulci (empty arrows). Note the marked dilatation of the right hypoglossal canal (arrowhead). (**e**) The sagittal T1-weighted image shows hypoplasia of the anterior portions of the corpus callosum (thick arrow). (**f**–**h**) Axial (**f**) and coronal (**g**,**h**) T2-weighted images reveal a normal cerebellum.

During hospitalization, the patient exhibited inverted sleep–wake rhythm and episodes of psychomotor agitation characterized by crying, screaming, head shaking movements, outward aggression and self-injurious behaviors (slaps, head-butting against the bed). At neurological evaluation, he showed no visual engagement or eye contact. While in the supine position, he transitioned into a seated posture and was able to crawl around the room. His gait was ataxic and unsteady. Brain and spine MRI was performed on a clinical 1.5T scanner according to standard protocols and showed microcephaly, a vertical splenium, and a small left cerebellar hemisphere with bilateral cerebellar foliation anomalies resulting in cerebellar dysplasia and vermian hypoplasia (Figure 4a–d). Mild hypoplasia of the pons was present. As an incidental finding, a markedly enlarged right hypoglossal canal was noted (Figure 4b).

Given the complex clinical presentation and multi-organ involvement, genetic consultation was undertaken, leading to the recommendation of CGH array and whole-exome sequencing. NGS revealed the heterozygous deletion of two nucleotides (TA) at positions 1648_1649 in the coding sequence of the *ASXL3* gene, which was confirmed by Sanger sequencing. Genetic testing was extended to the younger brother (proband 2), who carries the same mutation in heterozygosity, and to the mother, who was found to be mosaic for the variant.

### 3.2. Proband 2

In this case, the patient is a 20-month-old boy, who was born in Albania. He was delivered at 38 weeks of gestation via spontaneous vaginal delivery to a 34-year-old mother. Birth weight was 2900 g (16th percentile), while length and head circumference were not recorded. Fetal movements were reported to be reduced, although prenatal ultrasounds were reported as unremarkable. In addition to the neurodevelopmental concerns on the maternal side of the family, the paternal history was notable for nasopharyngeal carcinoma in the father, who also had a history of alcohol abuse and smoking. The paternal half-sister was reported to be in good general health, with normal neuropsychomotor development.

The patient suffered from neonatal jaundice and underwent surgery for inguinal hernia at 3 months of age. Medical history includes *Varicella-zoster* virus (VZV) infection at 9 months of age and recurrent febrile illnesses requiring antibiotic treatment. Delayed psychomotor development and growth retardation were also evident.

Clinical examination revealed impaired length and weight (respectively 77 cm and 8.050 kg, both < 2nd percentile according to CDC 2009 growth charts), normal head circumference (46.5 cm, 15th percentile), global hypotonia, and joint laxity, particularly at the hip joints.

Neurodevelopmental assessment revealed significant language impairment along with severe psychomotor and global developmental delay. The patient had not yet achieved independent ambulation and primarily mobilized by crawling. Pelvic X-ray showed normal hip joint alignment.

Concerning the recurrent daily vomiting, abdominal US was performed, and no pathological findings were found. The esophageal transit X-ray was normal, and dysphagia was excluded. Given the clinical features suggestive of gastroesophageal reflux disease, proton pump inhibitor therapy was initiated, resulting in symptom resolution.

Brain and spine MRI was performed on a clinical 3T scanner according to standard protocols and showed mild hypoplasia of the corpus callosum with a normal cerebellum (Figure 4e–h).

**Figure 5:**
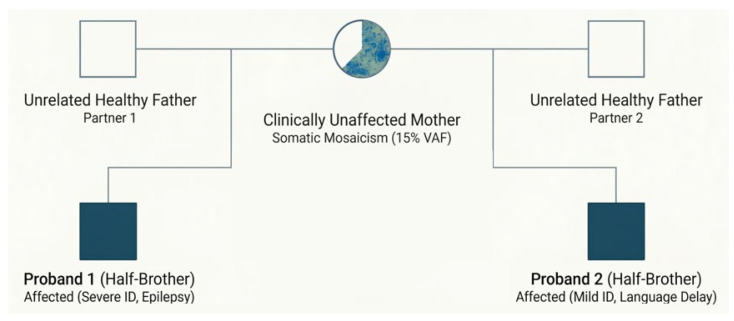
Familial transmission of BRS via maternal mosaicism.

CGH array analysis was normal. NGS revealed the presence of the same pathogenic variant in the *ASXL3* gene (NM_030632: c.1648_1649del) identified in the brother in heterozygous form and in the mother in mosaic form. The family pedigree is shown in Figure 5.

## 4. Discussion

Most published studies on Bainbridge–Ropers syndrome have described de novo mutations in the *ASXL3* gene, predominantly in unrelated individuals with only rare occurrences among siblings.

We report a family with inherited *ASXL3*-related disorder, consisting of two clinically affected half-brothers with a likely pathogenic heterozygous variant and their unaffected mother who carries the variant in mosaic form.

BRS represents a rare neurodevelopmental disorder whose diagnosis remains particularly challenging in clinical practice, mainly due to the broad and heterogeneous spectrum of neurodevelopmental conditions presenting with overlapping features. Indeed, global developmental delay and intellectual disability are highly non-specific and may underlie a wide range of genetic syndromes. The differential diagnosis is therefore complex and requires careful clinical and molecular evaluation. Although patients with BRS may exhibit a recognizable pattern of craniofacial dysmorphisms, the features are not pathognomonic, and they can be variably expressed. This diagnostic complexity underscores the importance of integrating phenotypic assessment with comprehensive genetic analysis in patients presenting with unexplained neurodevelopmental delay [28].

Whole-exome sequencing (WES) and Sanger sequencing were performed in both patients and identified a heterozygous deletion of two nucleotides (TA) at positions c.1648_1649 in the coding sequence of the *ASXL3* gene, which results in the substitution of methionine by aspartic acid at codon 550 and the introduction of a premature stop codon (p.Met550Aspfs*5). The variant is predicted to result in a truncated protein and is expected to have a loss-of-function effect. Although truncating mutations are commonly observed as causative of BRPS, this specific variant has not been previously reported in the literature, in public variant databases (ClinVar; National Center for Biotechnology Informatio, Bethesda, MD, USA; https://www.ncbi.nlm.nih.gov/clinvar/, accessed on 7 September 2025) or in the general population (Genome Aggregation Database, gnomAD; Broad Institute, Cambridge, MA, USA; https://gnomad.broadinstitute.org/, accessed on 7 September 2025). This suggests that it represents a novel pathogenic variant within the *ASXL3* gene mutational spectrum.

Molecular analysis was also performed in the patients’ mother, and it identified the pathogenic variant in mosaic form, with a variant allele fraction (VAF) of approximately 15% in peripheral blood DNA. Parental mosaicism has only rarely been reported in association with Bainbridge–Ropers syndrome. Early evidence suggesting germline mosaicism was provided by Koboldt et al., who described two affected sisters carrying the same de novo nonsense variant in the *ASXL3* gene despite negative parental testing in peripheral blood [8]. The authors proposed parental germline mosaicism as the most plausible explanation for the recurrence observed within the family. Subsequently, Schirwani et al. described a cohort of five individuals from three families with *ASXL3*-related disorders and identified inheritance patterns compatible with parental mosaicism [14]. In one patient, whole-exome sequencing revealed a variant allele fraction of approximately 30–35% in blood and saliva, suggesting post-zygotic mosaicism, while another variant was inherited from an apparently unaffected mother. More recently, Zhao et al. documented the first case of molecularly confirmed paternal mosaicism in BRPS using ultra-deep sequencing, detecting the pathogenic variant at a variant allele fraction of 8.17% in peripheral blood and 15.03% in sperm [15]. These observations indicate that mosaic variants may represent an underrecognized mechanism underlying the familial recurrence of *ASXL3*-related disorders. Our finding of molecularly confirmed maternal mosaicism further supports this hypothesis.

This report provides a detailed clinical description of two half-brothers affected by Bainbridge–Ropers syndrome (Table 1). The elder sibling was born preterm and small for gestational age (SGA) and experienced respiratory difficulties at birth. The other one displayed decreased fetal movement during the prenatal period and suffered from neonatal jaundice and recurrent infections.

Consistent with the literature, both patients showed joint laxity and craniofacial dysmorphism, including a prominent forehead, arched eyebrows, hypoplastic alae nasi, prominent columella and tooth malposition with dental overcrowding. A newly described feature was ptosis, observed in the patients and, interestingly, also in their mother. Specifically, ptosis was the only clinically appreciable physical feature observed in the mother.

From a neurodevelopmental perspective, both brothers presented with intellectual disability—severe in P1 and mild in P2—and a complete absence of verbal language. Hypotonia was noted in P2, while drug-resistant epilepsy, sleep disturbance and additional behavior manifestations, such as aggression, hand flapping and agitation, were observed in P1. Considering that language impairment is present in almost all patients with BRPS and that communication difficulties can lead to frustration and subsequent behavioral issues, providing access to tools that support nonverbal interaction is crucial.

Regarding sleep disturbances, polycomb group proteins are essential for circadian clock function and sleep homeostasis control [29]. ASXL3, a member of the polycomb regulatory machinery, exhibits peak cerebellar expression during early neurodevelopment, specifically up to postconception week 24, suggesting a critical role in establishing neural circuits governing arousal and sleep [30]. Furthermore, ASXL3 is expressed in key sleep–wake regulatory centers, including the hypothalamus: altered ASXL3-mediated chromatin regulation may therefore impair the development and function of multiple arousal systems, potentially contributing to the profound sleep–wake disturbances observed in BRS.

Neuroimaging abnormalities are observed in nearly 40% of individuals with Bainbridge–Ropers syndrome, including findings such as white matter loss, delayed myelination, ventriculomegaly, corpus callosum hypoplasia, cerebellar anomalies, and acquired brain lesions [13,25]. These findings are often non-specific and may be associated with epilepsy, as observed in P1. Among cerebellar anomalies, hypoplasia of the cerebellar vermis is the most frequently reported, whereas ponto-cerebellar hypoplasia has only been documented in a single individual with an *ASXL3* gene mutation [31]. In the present case (P1), we report cerebellar dysplasia with unilateral hemispheric hypoplasia, a rare neuroimaging feature previously described in only one individual with Bainbridge–Ropers syndrome [25]. This malformation is characterized by the underdevelopment of one cerebellar hemisphere along with abnormal foliation and is typically attributed to in utero vascular disruption during key stages of cerebellar development [32]. Similar imaging findings have been described in genetic conditions affecting vascular integrity, including COL4A1-related disorders and PHACE syndrome. Moreover, adverse intrauterine conditions—particularly maternal diabetes—have been implicated in the pathogenesis of cerebellar impaired development. We therefore hypothesize that the cerebellar abnormalities observed in Bainbridge–Ropers syndrome may result from pregnancy-related complications and/or a multifactorial interplay of genetic susceptibility, prenatal vascular insult, and environmental influences.

Both patients exhibited multi-organ involvement. The younger sibling experienced recurrent vomiting associated with gastroesophageal reflux disease and feeding difficulties. The elder one showed a colonic lesion detected by US and MRI, diagnosed as juvenile polyp upon histological examination. This feature has not been previously included in the currently known phenotypic spectrum of the syndrome and may represent an incidental finding.

Moreover, abdominal MRI incidentally revealed two renal cysts in the right kidney. Regarding this finding, two considerations can be made. First, this could be the first report describing renal cysts in *ASXL3*-related disorders, while urinary tract anomalies such as hydronephrosis and vesicoureteral reflux have been previously reported.

## 5. Conclusions

We report a case of familial Bainbridge–Ropers syndrome caused by a novel pathogenic variant of the *ASXL3* gene, identified in two clinically affected half-brothers and inherited from their clinically unaffected mother.

The frameshift variant (c.1648_1649del; p.Met550Aspfs*5) has not been previously reported in the literature, expanding the known mutational spectrum associated with *ASXL3*-related disorders.

The identification of newly described clinical features adds a valuable contribution to the clinical understanding of the syndrome. Additionally, this report highlights the first documented association of renal cysts and *ASXL3*-related disorders.

Overall, this report expands both the molecular and clinical spectrum of *ASXL3*-related disorders and underscores the importance of considering parental mosaicism during genetic evaluation. Increased awareness of mosaic variants and the application of sensitive molecular diagnostic approaches may improve the detection of low-level parental mosaicism and ultimately enhance genetic counseling and clinical management for affected families.

## Figures and Tables

**Table 1:** Clinical comparison of half-brothers with Bainbridge–Ropers syndrome.

	P1	P2
Age (years)	12	1.7
Sex (M/F)	M	M
Anthropometry (pc)		
Weight (percentile)	14th	<2nd
Length (percentile)	-	<2nd
OFC (percentile)	<2nd	15th
Perinatal		
Weeks at birth	36	38
Delivery	Normal	Normal
Weight (percentile)	<10th	16th
Respiratory difficulties	+	ND
Dysmorphic features		
Prominent forehead	+	+
Arched eyebrows	+	+
Hypoplastic alae nasi	+	+
Prominent columella	+	+
High palate	+	+
Tooth malposition	+	+
Skeletal		
Joint laxity	+	+
Gastrointestinal		
Feeding problems	-	+
GERD	-	+
Other	Juvenile colic polyp	-
Urinary tract	Kidney cysts	-
Ophthalmology		
Strabismus	+	+
Ptosis	+	+
Neurologic		
ID	Severe	Mild
Seizure	+	-
Hypotonia	-	+
Language impairment	+	+
Hand flapping	+	-
Sleep disturbance	+	-
Aggressive behavior	+	-
Agitation	+	-
Brain MRI findings	Hypoplasia of the corpus callosum	Minor midline dysmorphism

## Data Availability

The original contributions presented in this study are included in the article. Further inquiries can be directed to the corresponding author.

## Abbreviations

The following abbreviations are used in this manuscript:

BRPS	Bainbridge–Ropers syndrome
*ASXL3*	Additional sex combs-like 3
ASX	Additional sex combs
PR-DUB	Polycomb repressive deubiquitinase
BOPS	Bohring–Opitz syndrome
SHAPNS	Shashi–Pena syndrome
ID	Intellectual disability
ES	Exome sequencing
